# The influence of the doping concentration and reverse intersystem crossing on the efficiency of tricomponent organic light-emitting diodes with the thermally activated delayed fluorescence exciplex emitter

**DOI:** 10.1039/d4ra02394c

**Published:** 2024-06-18

**Authors:** Zhenyong Guo, Zhiqi Kou, Xiangqiong Xie, Yanbo Wang, Xinyu Zhu, Qixuan Jin, Chenchen Wang

**Affiliations:** a College of Science, University of Shanghai for Science and Technology Shanghai China usst102@aliyun.com

## Abstract

In this work, we fabricate a series of full-fluorescent organic light-emitting diodes (OLEDs) with the thermally activated delayed fluorescence (TADF) exciplex emitter in order to improve the efficiency through the reverse intersystem crossing (RISC) process. The TADF exciplex emitters are made up of a mixture of P-type materials (DMAC-DPS and mCBP) and n-type material (PO-T2T), among which DMAC-DPS also classes as a TADF material. The change in doping concentration will affect the intermolecular distance and the composition of TADF material and two kinds of exciplexes (DMAC-DPS:PO-T2T and mCBP:PO-T2T) in the luminescent layer (EML). Different materials and concentrations of doping not only add new RISC channels but also alter the original RISC channels, thereby affecting the performance of devices. It is beneficial for improving efficiency by increasing the proportion of independent TADF material and reducing the proportion of exciplex (DMAC-DPS:PO-T2T) in the EML, which can be controlled by doping. When the ratio of DMAC-DPS, PO-T2T and mCBP in the EML is 1 : 1 : 2, we achieve the optimal electro-optic performance in device A3, with maximum current efficiency, power efficiency, and luminance of 41.64 cd A^−1^, 43.42 lm W^−1^, and 23 080 cd m^−2^, respectively.

## Abstract 2

We fabricate a series of fluorescent organic light-emitting diode (OLED) with the thermally activated delayed fluorescence (TADF) exciplex emitter in order to improve efficiency through the reverse intersystem crossing (RISC) process.

## Graphical abstract

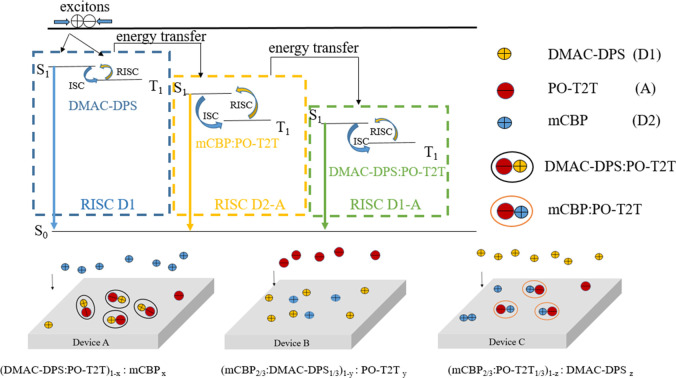

## 1. Introduction

Over the past few years, organic light-emitting diodes (OLEDs) have shown great potential for next-generation flat-panel displays and solid state lighting (SSL) due to their excellent performance in terms of high brightness, wide viewing angle, fast response time, and low power consumption.^1–4^ For OLEDs based on conventional fluorescent materials (FOLEDs), the maximum internal quantum efficiency (IQE) is limited to 25% as only singlet excitons can be utilized.^5^ Although 100% IQE can be realized in OLEDs based on phosphorescent materials (PhOLEDs) because they can simultaneously capture excitons in both singlet and triplet excitons, the high costs and unclear toxicities of precious-metal materials always limit their further development.^6–9^ In recent years, non-conventional fluorescent materials such as the thermally activated delayed fluorescence (TADF) material and exciplex forming co-hosts have attracted tremendous attention and effort from both academia and industry.^9,10^ Among them, low-energy triplet excitons can obtain energy to up-convert to the emissive singlet level through the endothermic reverse intersystem crossing (RISC) process.^11^

In 2012, Adachi *et al.* reported a class of metal-free organic electroluminescent molecules in which the energy gap between the singlet and triplet excited states was minimized by design, and these simple aromatic compounds exhibited efficient TADF with high photoluminescence efficiency.^12^ Adachi and co-workers also first demonstrated the TADF characteristics of exciplexes and achieved an external quantum efficiency (EQE) of 5.4% by using a blend exciplex (m-MTDATA:3TPYMB) as emitter.^13^ Compared with single-molecule TADF emitters, TADF exciplex emitters do not need complicated molecule design and synthesis, and just require suitable electron-donating (D) and electron-accepting (A) constituent molecules.^9^ Lee *et al.* presented a practical and straightforward approach to improve the emission performance of the TADF type exciplex by dispersing the exciplex (DMAC-DPS:PO-T2T) in the TADF host (DMAC-DPS). The EQE of the exciplex device was improved to 15.3% by adopting the emitting layer (EML) structure with low n-type material (PO-T2T) content because of the suppressed exciton quenching by spatial separation of the exciplex in EML.^14^ In this article, the author used three materials to create two RISC channels. Zhang *et al.* proposed a strategy of building TADF exciplex emitters with three components to realize three RISC channels respectively on DBT-SADF, DBT-SADF:PO-T2T, and CDBP:PO-T2T, which can effectively improve the exciton utilization in TADF exciplex emitters.^15^ The author attributed the increased RISC process to their reduced energy gap (Δ*E*_ST_), which can be realized by separating the spatial distance of the electron–hole pairs. However, they did not provide a detailed discussion on the proportion of three components in EML and the role of each RISC channel.

In our work, we propose a simple approach to realize three RISC channels in the full-fluorescent OLEDs with TADF exciplex emitter by doping and investigate the relationship between RISC channels and efficiency. 3,3-Di(9*H*-carbazol-9-yl)biphenyl (mCBP) are used as the p-type molecules, 10,10′-(sulfonylbis(4,1-phenylene))bis(9,9-dimethyl-9,10-dihydroacridine) (DMAC-DPS), a well-known TADF material,^16,17^ the n-type molecule is 2,4,6-tris[3-(diphenylphosphinyl)phenyl]-1,3,5-triazine (PO-T2T).^18^ RISC channels can be achieved in both TADF material (DMAC-DPS) and two kinds of exciplexes materials (DMAC-DPS:PO-T2T and mCBP:PO-T2T). We investigate the influence of the doping concentration on the change of RISC channels, which can be achieved through sequentially doping three materials (mCBP, PO-T2T and DMAC-DPS) into different binary systems (devices A1, B1 and C1), respectively. Moreover, the relationships between RISC channel changes and changes in electro-optical and spectral performance are also discussed in detail. When the ratio of DMAC-DPS, PO-T2T and mCBP in EML is 1 : 1 : 2, we achieve the optimal electro-optic performance in device A3, with maximum current efficiency, power efficiency, and luminance of 41.64 cd A^−1^, 43.42 lm W^−1^, and 23 080 cd m^−2^, respectively. The normalized electroluminescence (EL) spectrum of device A3 remains almost unchanged as the voltage changes from 3.5 V to 11.5 V.

## 2. Experimental

The indium-tin oxide (ITO) layer is precoated onto the glass substrate. The glass substrates with a surface resistivity of 15 Ω sq.^−1^ are sequentially cleaned with cleaning agent solution, deionized water, ethanol, and isopropanol, each step lasting for 10 minutes, followed by drying and cooling in a drying oven for 30 minutes. All devices are prepared by vacuum deposition at a pressure below 5 × 10^−4^ Pa. During the fabricating process, all organic materials are grown on substrates at a rate of 0.01–1 Å s^−1^. The evaporation rate of the doping material is adjusted based on the doping concentration. The electro-optical characteristics and EL spectra of all devices are measured by using a Keithley 2400 source meter and a PR655 spectroradiometer, respectively. All the measurements are carried out in the ambient atmosphere.

**Fig. 1:**
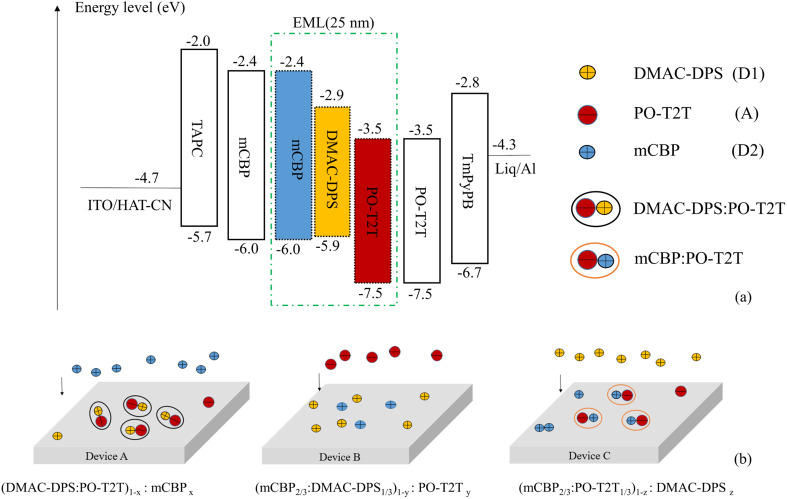
(a) The energy level diagrams of all materials used in our tricomponent OLEDs; (b) schematic diagrams in EML of fabricated devices A, B and C by doping.

The energy level diagrams of all materials used in our tricomponent OLEDs with the TADF exciplex emitter and the schematic diagrams in EML of devices A, B and C are shown in Fig. 1. In our experiments, 1,4,5,8,9,11-hexaazatriphenylene-hexacarbonitrile (HAT-CN) and 8-hydroxyquinolinolato lithium (Liq) serve as the hole injection layer (HIL) and the electron injection layer (EIL).^19,20^ The 1,1-bis (di-4-tolylaminophenyl) cyclohexane (TAPC) and mCBP act as the hole transport layer (HTL).^21^ PO-T2T and 1,3,5-tri(*m*-pyrid-3-ylphenyl)benzene (TmPyPB) act as the electron transport layer (ETL).^22^ All the highest occupied molecular orbital (HOMO) and the lowest unoccupied molecular orbital (LUMO) energy level of all organic materials are obtained from the representative works.^17–22^ The basic structure of all devices is as follows: ITO/HAT-CN (10 nm)/TAPC (40 nm)/mCBP (6 nm)/EML (25 nm)/PO-T2T (10 nm)/TmPyPB (40 nm)/Liq (1 nm)/Al (100 nm). The tricomponent EML is composed of three component materials DMAC-DPS, PO-T2T and mCBP in different proportions, respectively.

## 3. Results and discussion

**Fig. 2:**
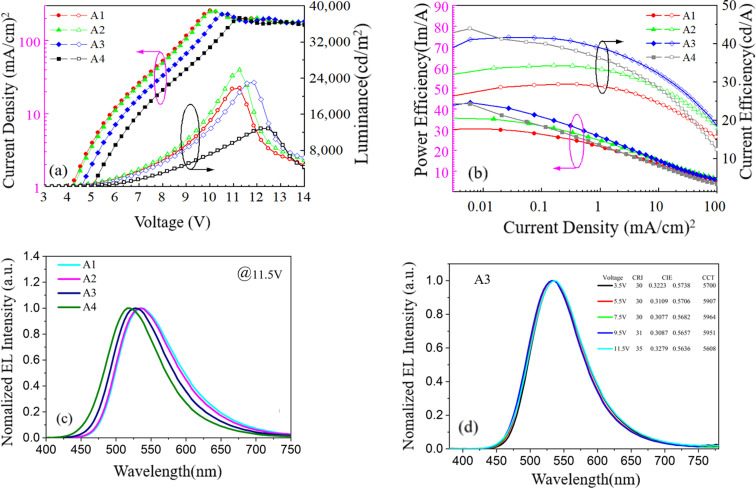
(a) Current density and luminance *versus* voltage curves of devices A1–A4; (b) power efficiency and current efficiency *versus* current density curves of devices A1–A4; (c) the normalized EL spectra of devices A1–A4 at 11.5 V driving voltage; (d) the normalized EL spectra of device A3 at different voltages from 3.5 V to 11.5 V.

Firstly, we investigate the effect of the doping concentration (*x*) of p-type material mCBP in EML on electro-optical and spectral characteristics in series of device A, which is based on device A1 with a TADF exciplex emitter (DMAC-DPS:PO-T2T). As indicated in Fig. 1(b), the values of *x* in devices A1–A4 varies, corresponding to 0%, 25%, 50% and 75%, respectively. Fig. 2(a) shows the luminance–voltage–current density characteristic curves of devices A1–A4. Device A2 achieves the maximum luminance of 25 830 cd m^−2^ among all four devices, but its efficiencies are lower than that of devices A3 and A4 as shown in Fig. 2(b) and Table 1. As the doping concentration of mCBP increases, the current efficiency and power efficiency of the device are gradually improving. Although the device A4 with the highest doping concentration of 75% has a maximum current efficiency of 42.55 cd A^−1^ and a maximum power efficiency of 43.95 lm W^−1^, its maximum luminance is only 12 880 cd m^−2^. In device A3, the efficiency and luminance of the device are relatively high, with the current efficiency, power efficiency, and luminance being 41.64 cd A^−1^, 43.42 lm W^−1^, and 23 080 cd m^−2^, respectively. The detailed values of EL characteristic for all devices tested with different structures are listed in Table 1.

**Table 1:** Electro-optical characteristics of all devices tested with different structures

Device	CD_max_^a^ (mA cm^−2^)	*L*_max_ b (cd m^−2^)	CE_max_^c^ (cd A^−1^)	PE_max_^d^ (lm W^−1^)	CIE_(*x*,*y*)_@3.25 V	CIE_(*x*,*y*)_@11.5 V	EQE (%)
A1	260.01	21 820	28.59	29.94	0.382, 0.571	0.370, 0.566	9.26
A2	252.53	25 830	33.67	35.26	0.365, 0.571	0.371, 0.566	10.64
A3	232.73	23 080	41.64	43.42	0.327, 0.574	0.328, 0.564	12.42
A4	204.85	12 880	42.55	43.95	0.278, 0.547	0.287, 0.536	13.77
B1	181.72	4325	29.00	28.04	0.279, 0.529	0.267, 0.499	9.38
B2	207.94	17 570	26.20	27.44	0.408, 0.559	0.386, 0.563	8.60
B3	193.02	9040	22.85	23.93	0.403, 0.559	0.372, 0.564	7.23
C1	154.65	3227	21.76	21.03	0.284, 0.505	0.236, 0.441	7.60
C2	169.26	11 120	31.79	33.29	0.314, 0.575	0.309, 0.561	9.99
C3	179.44	6149	20.70	20.01	0.295, 0.559	0.302, 0.548	6.45

a The maximum current density.

b The maximum luminance.

c The maximum current efficiency.

d The maximum power efficiency.

**Fig. 3:**
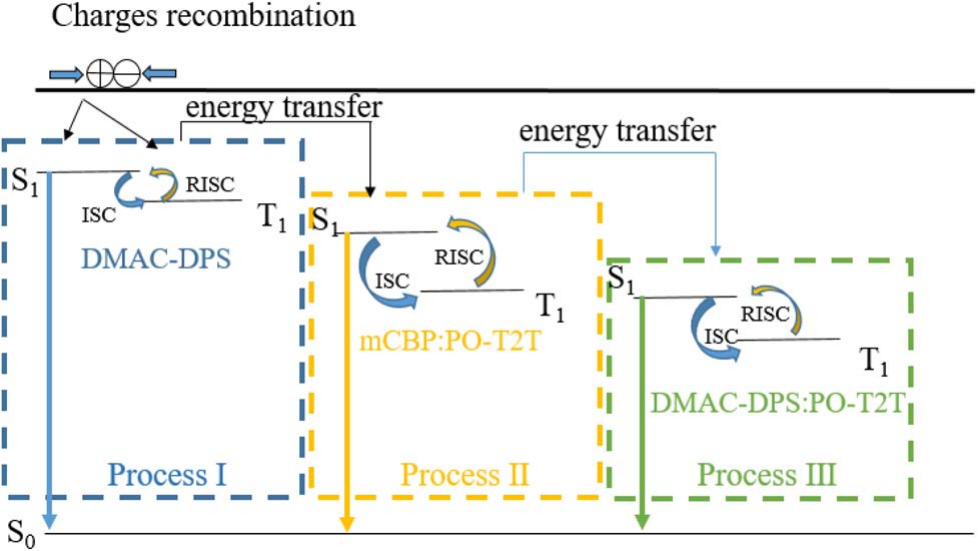
Schematic diagram of energy transfer including RISC channels in EML of device A, B and C.

The relative spatial distance between donors and acceptors in exciplex systems can affect the potential energy surfaces of ground states and excited states, which in turn affects the emission characteristics of the device.^15,23^ Yuan *et al.* improve electro-optical characteristics of TCTA:PO-T2T exciplex-based OLEDs by co-doping an organic spacer (mCP) into the bulk-heterojunction exciplex emitter (TCTA:PO-T2T) because of the long-range coupling of the electron–hole pairs. They believe that the increased RISC process from the triplet to singlet states is due to their reduced energy gap (Δ*E*_ST_).^15,24,25^ Besides the reduced Δ*E*_ST_, we regard that the changes of the RISC channel caused by doping and dispersing molecules are also another important reason for the remarkable improvement in device performance. As shown in Fig. 3, there are only two RISC channels (process I and process III) resulting from TADF material DMAC-DPS and exciplex material DMAC-DPS:PO-T2T in device A1, respectively. The doping of p-type material mCBP increases the distance between molecules DMAC-DPS and PO-T2T and makes their density more dispersed in EML, which reduces the probability of forming exciplex (DMAC-DPS:PO-T2T). The energy level of process II is between that of process I and process III.^26–28^ As the doping concentration of mCBP increases, the probability of forming the independent TADF material (DMAC-DPS) and the exciplex (mCBP:PO-T2T) in EML increases significantly, which results in an increase in the number of excitons involved in the first and the second RISC channels (process I and process II). Improving device efficiency through process I and process II is more effective than through process III. So device A4 achieves the maximum current and power efficiency of 42.55 cd A^−1^ and 43.95 lm W^−1^, respectively. Because the HOMO energy level of mCBP is higher than that of DMAC-DPS as shown in Fig. 1(a), the excessive mCBP doping makes the current injection more difficult. The current density of device A4 gradually decreases compared to that of device A3, resulting in a decrease in luminance from 23 080 cd m^−2^ to 12 880 cd m^−2^.

Fig. 2(c) indicates the normalized EL spectra of devices A1–A4 at 11.5 V voltage. In device A1 without mCBP doping, the main peak wavelength of the EL spectrum at 536 nm mainly comes from the energy release of the singlet energy level (2.32 eV) of the exciplex material DMAC-DPS:PO-T2T.^26^ The TADF material DMAC-DPS has a singlet energy level of 2.9 eV, corresponding to the main peak position of 520 nm in the EL spectra.^27^ As the doping concentration increases, the spectral peak values of devices A1–A4 exhibit a slight blue shift, with the color coordinate ranging from (0.370, 0.566) to (0.287, 0.536). This blue shift indicates that the change in the ratio of exciplexes and TADF material has led to a shift in the main RISC process of excitons from process I and process III to process I and process II. As the voltage changes from 3.5 V to 11.5 V, the spectral variation of device A3 is little as shown in Fig. 2(d).

**Fig. 4:**
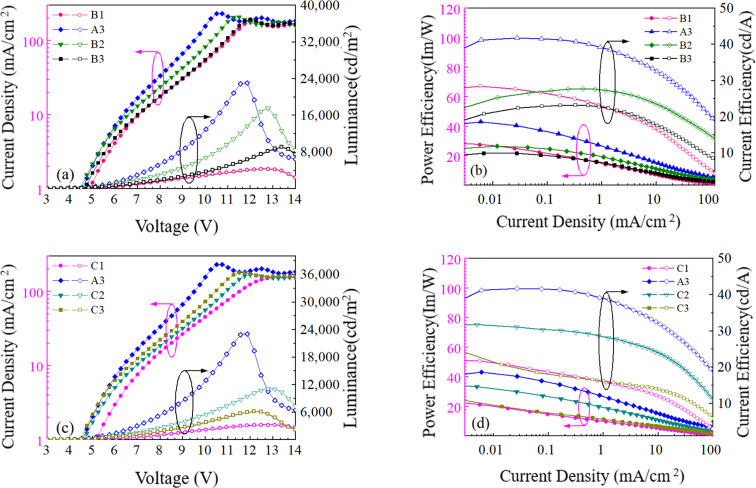
(a) Current density and luminance *versus* voltage curves of devices B1, A3, B2 and B3; (b) power efficiency and current efficiency *versus* current density curves of devices B1, A3, B2 and B3; (c) current density and luminance *versus* voltage curves of devices C1, A3, C2 and C3; (d) power efficiency and current efficiency *versus* current density curves of devices C1, A3, C2 and C3.

**Fig. 5:**
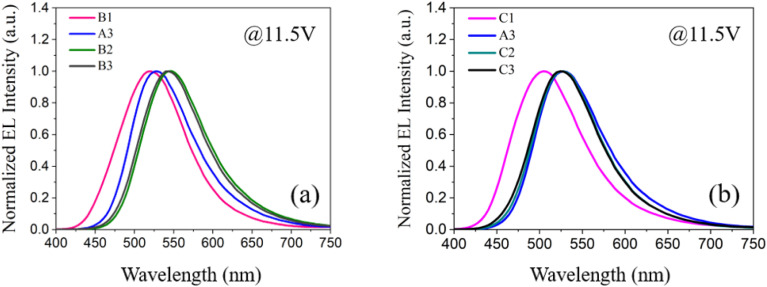
(a) The normalized EL spectra of devices B1, A3, B2 and B3 at 11.5 V driving voltage; (b) the normalized EL spectra of devices C1, A3, C2 and C3 at 11.5 V driving voltage.

Secondly, we investigate the effect of the doping concentration (*y*) of the n-type material PO-T2T on the electro-optical and spectral characteristics of device B prepared on the basis of device A3. As indicated in Fig. 1(b), the values of *y* in devices B1, A3, B2 and B3 varies, corresponding to 0%, 25%, 50% and 75%, respectively. Fig. 4(a), (b) and Table 1 show the electro-optical characteristic curves and experimental data of the devices B1, A3, B2 and B3. Compared with three B devices, device A3 has the highest maximum current efficiency, power efficiency, and luminance, with values of 41.64 cd A^−1^, 43.42 lm W^−1^, and 23 080 cd m^−2^, respectively. In device B1, there is only a unique RISC channel (process I) resulting from TADF material DMAC-DPS in EML, as there is no exciplex due to the concentration of PO-T2T being 0. As the doping concentration of PO-T2T increases, the distance between PO-T2T molecules and DMAC-DPS and mCBP molecules also decreases, which makes it easier to form exciplex mCBP:PO-T2T and DMAC-DPS:PO-T2T. As the content of PO-T2T gradually increases from 0, the RISC channel in process II and process III begins to appear. At low doping concentration, multiple RISC channels can cause more excitons to up-convert to the emissive singlet state of TADF material, thereby improving the maximum power efficiency to 43.42 lm W^−1^ of device A3. However, as the doping content of PO-T2T further increases, the probability of forming exciplex (DMAC-DPS:PO-T2T) increases and the probability of the independent TADF material decreases significantly. This will significantly weaken the role of RISC channel of process I, and the maximum power efficiency of the device will begin to decrease to 23.93 lm W^−1^ of device B3. At the same time, the position of the main peak in the normalized EL spectra shifts from (0.267, 0.499) to (0.372, 0.564) with increasing the doping concentration of PO-T2T as shown in Fig. 5(a). This also indicates that the luminescence resulting from two exciplexes (mCBP:PO-T2T, DMAC-DPS:PO-T2T) has become the main source process. The luminescence of TADF material almost disappears in devices B2 and B3.

Finally, we investigate the effect of the doping concentration (*z*) of the TADF material DMAC-DPS on the electro-optical and spectral characteristics of device C prepared on the basis of device A3. As indicated in Fig. 1(b), the values of *z* in devices C1, A3, C2 and C3 varies, corresponding to 0%, 25%, 50% and 75%, respectively. Fig. 4(c), (d) and Table 1 show the electro-optical characteristic curves and experimental data of the devices C1, A3, C2 and C3. Compared with device C, the maximum current, power efficiency, and luminance in device A3 are still the highest among all devices. In device C1, there is only a unique RISC channel (process III) resulting from exciplex material mCBP:PO-T2T in EML, as there is no TADF material DMAC-DPS due to the concentration of DMAC-DPS being 0. When a small amount of DMAC-DPS is doped, the independent DMAC-DPS molecules and the exciplex material DMAC-DPS:PO-T2T coexist in EML because some DMAC-DPS and PO-T2T molecules still have a relatively large distance between them. As the doping content of DMAC-DPS increases, the average distance between DMAC-DPS and PO-T2T molecules further decreases, which results in a gradual decrease in independent TADF materials and an increase in exciplex material DMAC-DPS:PO-T2T. In Table 2, we list and compare the efficiencies of several OLEDs with the same exciplex (DMAC-DPS:PO-T2T), and find that our device A3 exhibits the better performance.

**Table 2:** Comparison of the efficiency with other reported OLED with the same exciplex

Device	CD_max_^a^ (mA cm^−2^)	*L*_max_ b (cd m^−2^)	CE_max_^c^ (cd A^−1^)	PE_max_^d^ (lm W^−1^)	CIE_(*x*,*y*)_@3.25 V	CIE_(*x*,*y*)_@11.5 V	EQE (%)
A3	232.73	23 080	41.64	43.42	0.327, 0.574	0.328, 0.564	12.42
Ref. 14	—	—	28.60	—	—	—	10.80
Ref. 29	—	—	38.10	25.30	—	—	12.11
Ref. 30	—	—	42.96	35.02	—	—	16.68

a The maximum current density.

b The maximum luminance.

c The maximum current efficiency.

d The maximum power efficiency.

Both DMAC-DPS doping and PO-T2T doping have the same effect, reducing the average distance between DMAC-DPS and PO-T2T. The corresponding effect of process I also weakens, while that of process II and III gradually strengthens. Therefore, the device efficiencies of devices B and C show a significant decrease compared to that of device A3 as high concentration doping occurs. A gradual red shift of the normalized EL spectra corresponding to a shift in exciton energy from the singlet energy level of DMAC-DPS to that of DMAC-DPS:PO-T2T is observed as the doping concentration (*z*) of DMAC-DPS increased from 0% to 75% as shown in Fig. 5(b).

## 4. Conclusion

In summary, we realize a change of the energy transfer path in a series of tricomponent OLEDs by doping different materials into TADF exciplex emitters. We ultimately achieve the best device performance in device A3, with maximum current efficiency, power efficiency, and luminance of 41.64 cd A^−1^, 43.42 lm W^−1^, and 23 080 cd m^−2^, respectively. These electro-optical characteristics far exceed the performance of conventional full-fluorescent OLEDs device. When low doping content in devices A, B, and C, the utilization efficiency of excitons in EML is improved due to the increase of RISC channels, thereby improving the device efficiency. When the doping concentration is greater than 25% in device B and C, the number of independent TADF molecules in EML decreases due to the decrease in intermolecular distance, and the RISC channel caused by TADF material weakens, resulting in a significant decrease in efficiency of devices B and C. The proportion between TADF material (DMAC-DPS) and two kinds of exciplexes (DMAC-DPS:PO-T2T and mCBP:PO-T2T) is closely related to the channels of RISC, and different channels affect the utilization efficiency of excitons in EML. The spectral shift with doping concentration in devices A, B and C is consistent with the change of RISC channels.

## Conflicts of interest

The authors declare that none of the work reported in this study has been influenced by any known financial or personal relationships.
